# Potential Predictors of Plasma Fibroblast Growth Factor 23 Concentrations: Cross-Sectional Analysis in the EPIC-Germany Study

**DOI:** 10.1371/journal.pone.0133580

**Published:** 2015-07-20

**Authors:** Romina di Giuseppe, Tilman Kühn, Frank Hirche, Brian Buijsse, Jutta Dierkes, Andreas Fritsche, Rudolf Kaaks, Heiner Boeing, Gabriele I. Stangl, Cornelia Weikert

**Affiliations:** 1 Research Group Cardiovascular Epidemiology, German Institute of Human Nutrition Potsdam-Rehbrücke, Nuthetal, Germany; 2 Division of Cancer Epidemiology, German Cancer Research Center (DKFZ), Heidelberg, Germany; 3 Institute of Agricultural and Nutritional Sciences, Human Nutrition Group, Martin Luther University Halle-Wittenberg, Halle (Saale), Germany; 4 Department of Epidemiology, German Institute of Human Nutrition Potsdam-Rehbrücke, Nuthetal, Germany; 5 Department of Clinical Medicine, University of Bergen, Bergen, Norway; 6 Department of Internal Medicine IV, University of Tübingen, Tübingen, Germany; 7 German Center for Diabetes Research (DZD e.V.), Neuherberg, Germany; 8 Institute for Social Medicine, Epidemiology and Health Economics, Charité University Medical Center, Berlin, Germany; 9 German Centre for Cardiovascular Research (DZHK), Partner Site Berlin, Berlin, Germany; University of Florida, UNITED STATES

## Abstract

**Background:**

Increased fibroblast growth factor 23 (FGF23), a bone-derived hormone involved in the regulation of phosphate and vitamin D metabolism, has been related to the development of cardiovascular disease (CVD) in chronic kidney disease patients and in the general population. However, what determines higher FGF23 levels is still unclear. Also, little is known about the influence of diet on FGF23. The aim of this study was therefore to identify demographic, clinical and dietary correlates of high FGF23 concentrations in the general population.

**Methods:**

We performed a cross-sectional analysis within a randomly selected subcohort of the European Prospective Investigation into Cancer and Nutrition (EPIC)-Germany comprising 2134 middle-aged men and women. The Human FGF23 (C-Terminal) ELISA kit was used to measure FGF23 in citrate plasma. Dietary data were obtained at baseline via validated food frequency questionnaires including up to 148 food items.

**Results:**

Multivariable adjusted logistic regression showed that men had a 66% lower and smokers a 64% higher probability of having higher FGF23 (≥ 90 RU/mL) levels compared, respectively, with women and nonsmokers. Each doubling in parathyroid hormone, creatinine, and C-reactive protein was related to higher FGF23. Among the dietary factors, each doubling in calcium and total energy intake was related, respectively, to a 1.75 and to a 4.41 fold increased probability of having higher FGF23. Finally, each doubling in the intake of iron was related to an 82% lower probability of having higher FGF23 levels. Results did not substantially change after exclusion of participants with lower kidney function.

**Conclusions:**

In middle-aged men and women traditional and non-traditional CVD risk factors were related to higher FGF23 concentrations. These findings may contribute to the understanding of the potential mechanisms linking increased FGF23 to increased CVD risk.

## Introduction

Fibroblast growth factor 23 (FGF23) is a phosphatonin involved in the regulation of phosphate, calcium, parathyroid hormone and vitamin D metabolism [1]. Higher FGF23 levels have been related to the development of cardiovascular disease (CVD) in chronic kidney disease patients [2]. Furthermore, in patients undergoing coronary angiography circulating FGF23 concentrations have been related to the severity and extent of coronary artery stenosis in the coronary angiographic patients [3]. However, FGF23 recently emerged as a non-traditional CVD risk factor also in the general population with normal renal function [4]. In both human [5] and experimental studies [6] higher FGF23 levels have been related to left ventricular hypertrophy (LVH). Since LVH is an independent risk factor, among others, for coronary heart disease (CHD), heart failure and stroke [7] epidemiologic studies have investigated possible relationships between FGF23 and major CVD endpoints in the general population. In particular, significant associations between higher FGF23 concentrations and increased risk of heart failure [8–13], total [8, 9, 14, 15] and hemorrhagic stroke [16, 17] myocardial infarction [17], CHD [11], all cause [9] and cardiovascular mortality [11, 13] emerged. These findings were recently confirmed in a meta-analysis of prospective cohort studies showing increased FGF23 levels related to an increased risk of all-cause mortality, CVD events, cardiovascular mortality, stroke, heart failure [18].

So far, less is known about factors that can modulate the plasma concentration of FGF23 in the general population.

Previous studies have shown associations between higher FGF23 levels and smoking, dyslipidemia, obesity [8, 19, 20], as well as higher age, dietary phosphorus intake, history of hypertension, and higher levels of PTH, serum phosphate, triglycerides, uric acid, interleukine-6, vascular cell adhesion molecule-1, and soluble tumor necrosis factor receptor 1 [21]. However, these relationships were observed in elderly [19, 20] and male subjects [20, 21], and in patients with stable coronary artery disease [8]. It remains unknown if these findings are generalizable to different populations and settings. Besides, little is known about the influence of diet on FGF23. Increased FGF23 could be the consequence of a prolonged exposure to high dietary phosphorus levels [22]. Yet, whether a low-phosphorus diet can prevent FGF23 elevation is unknown since available evidence are inconsistent and unlikely conclusive [23].

In the present study we aimed to identify demographic, clinical and dietary correlates of FGF23 and of high FGF23 concentrations in the general population. For this purpose we performed a cross-sectional analysis of the European Prospective Investigation into Cancer and Nutrition (EPIC)-Germany comprising middle-aged men and women.

## Methods

### Study population and design

EPIC-Germany comprises two cohorts at the study centres in Potsdam (27,548 participants: 16,644 women and 10,904 men; 5th and 95th age percentiles: 36–64) and Heidelberg (25,543 participants: 13,614 women and 11,929 men; 5th and 95th age percentiles: 37–63). Participants were recruited from the local general populations between 1994 and 1998 [24, 25].

For the present analyses a cross-sectional study design was chosen. The study sample consisted of a subcohort randomly selected from the original EPIC-Germany cohort [26] (n = 2172 participants).

### Ethics Statement

Study procedures have been approved by the Ethics Committee of the Medical Association of the State of Brandenburg and by the Ethics Committee of the Heidelberg University Medical School, respectively for Potsdam and Heidelberg. All participants gave their written informed consents prior their inclusion in the study.

### Exposure and covariate measurements

At baseline, a blood sample was drawn, processed and stored in liquid nitrogen at −196 C [25, 27]. In 2011, FGF23, PTH, and 25-hydroxyvitamin D3 [25(OH)D3] were measured in the Institute of Agricultural and Nutritional Science, University of Halle, Germany. The Human FGF23 (C-Terminal) enzyme-linked immunosorbent assay (ELISA) kit (Immutopics, San Clemente, CA, USA) was used to measure FGF23 concentrations in citrate plasma [28]. Our choice to measure the C-Terminal FGF23 rather than the intact FGF23 was driven by the significant diurnal variation of the latter, considering that a stable concentration during the entire day [29] and over time [28], as shown for the C-Terminal FGF23, is a prerequisite of a reliable marker.

Plasma PTH (intact) was measured by an ELISA Kit (Biomerica, Irvine, CA, USA). The plasma concentration of 25(OH)D3 was analysed by LC-MS/MS as described previously [30, 31]. The lower limit of quantification (LOQ) was 10 RU/mL, 10 pg/mL, and 4.6 ng/mL respectively for FGF23, PTH, and 25(OH)D3.

In Potsdam high-density lipoprotein cholesterol (HDL), total cholesterol and C-reactive protein (CRP) were measured with an automatic analyzer (ADVIA 1650, Siemens Medical Solutions, Erlangen, Germany) in 2007 at the Department of Internal Medicine of the University of Tübingen, Germany, while plasma creatinine was measured in 2008. In Heidelberg plasma creatinine was measured at the Stichting Huisartsen Laboratorium (Breda, The Netherlands). The Chronic Kidney Disease Epidemiology Collaboration equation (CKD-EPI) was used to estimate glomerular filtration rate (eGFR) [32]. To account for citrate’s dilution factor biomarkers’ concentrations were multiplied by 1.1.

Lifestyle characteristics, including sports activity and smoking history were documented at baseline by trained interviewers during PC-guided interview [24]. Total physical activity was assessed with the Cambridge Physical Activity Index [33]. In particular, this index combines self-reported occupational activity, categorized as sedentary, standing, manual, or heavy manual occupation, with time participating in cycling and sports. The sum of hours per week spent on cycling and sports were categorized in four levels and participants divided into four mutually exclusive categories, i.e. inactive (sedentary job and no recreational activity), moderately inactive, moderately active, and active (sedentary job with >1 h recreational activity per day, standing or physical job with some recreational activity, or a heavy manual job). Education was categorized into none, vocational school or less, technical school, secondary school, and university. Prevalent hypertension, hyperlipidemia, and diabetes were self-reported at baseline.

#### Anthropometric measures

Weight and height were measured with participants not wearing shoes. In Heidelberg, waist circumference was measured at the narrowest circumference of the torso, while in Potsdam at the midpoint between the lower ribs and iliac crest. Hip circumference was measured over the buttocks. To reduce heterogeneity due to protocol differences corrected measures of each participant’s body weight, waist and hip circumference were used. Body mass index (BMI) was defined as weight in kg divided by the square of the height in meters (kg/m^2^).

#### Dietary information

Dietary information was obtained with a food frequency questionnaire (FFQ) in the baseline assessment of EPIC-Germany, including up to 148 food items [25]. The optical readable FFQ was designed to capture the usual food and nutrient intake of individuals during the past 12 months.

### Statistical analysis

Right skewed variables were natural log-transformed. Analysis of covariance was used to compare the general characteristics including selected biomarkers according to quartiles of FGF23 concentrations.

FGF23, PTH and 25(OH)D3 values below or above the limit of quantification (LOQ) were substituted with the LOQ and a multivariable multiple imputation was used to impute missing values for the remaining variables [34] (S1 Table for the amount of missing data). SAS PROC MI with the Markov Chain Monte Carlo method for arbitrary missing patterns was used to generate 20 imputed datasets. The logarithmic transformation was taken for all the continuous right skewed variables. SAS PROC MIANALYZE was used to combine the results of the analyses on 20 imputed datasets and to generate valid statistical inferences.

To identify FGF23 correlates a regression analysis based on the best subsets regression procedure was applied, with FGF23 concentrations as dependent variable and two categories of independent variables (dietary and non-dietary covariates). Independent variables were selected based on a priori knowledge: age, sex, BMI, waist circumference, prevalent hypertension, hyperlipidemia, diabetes, and cardiovascular disease, educational attainment, physical activity, smoking status, parathyroid hormone, 25(OH)D3, creatinine, total and HDL-cholesterol, C-reactive protein (CRP), season of blood draw, intakes of total dietary fat, carbohydrate, protein and fibre, phosphorus, calcium, potassium, magnesium, iron, alcohol, energy, and use of calcium supplements. Both physical activity and dietary total protein intake were categorized into quartiles to take nonlinear relationships into account. To estimate the inorganic phosphorus intake as a potential predictor of FGF23 we calculated the ratio of phosphorus to total protein intake as this ratio reflects the amount of inorganic phosphorus from food additives best [35].

The best subsets regression procedure was performed on each multiply imputed dataset. FGF23 correlates were kept in the final model, chosen based on the Mallow's *C*_*p*_-statistic and Akaike‘s Information criterion, as identified in each multiply imputed dataset. A multivariable linear regression analysis was then used to investigate the relations between plasma FGF23 and the identified best set of correlates. Furthermore, a logistic regression model was performed to determine the correlates associated with higher FGF23 levels using a cut-off point of 90 RU/mL (n = 269) [16, 17]. Results were expressed as Odds Ratio (OR) and 95% confidence interval (95% CI). For a better interpretation of the results, both biomarkers and dietary data were log base 2 transformed and results interpreted as OR per 2-fold increase in predictors.

Analyses were performed with SAS 9.4 (SAS Institute, Cary, NC). Statistical tests were two-sided and P values <0.05 considered statistically significant.

### Exclusion criteria

38 participants in the lowest and highest first percent distribution of the ratio of total energy intake to energy requirement were excluded from the analyses, leaving a final sample of 2,134 subjects.

### Sensitivity analysis

Analyses were repeated after exclusion of 73 participants with eGFR <60 mL/min/1.73m^2^ due to concern of renal function-related increase in FGF23 concentrations.

## Results

Of the study population (n = 2,134) 44.1% were male and 55.9% were female with a median age of 51.7 years, interquartile range [(IQR): 43.7–58.3)]. Overall, median FGF23 concentrations were 53.7 RU/mL, IQR: 39.9–71.5. Tables 1 and 2 show, respectively, demographic, lifestyle, clinical, and dietary characteristics of the EPIC-Germany participants by quartiles of FGF23concentrations. Individuals with higher FGF23 levels (median in the highest FGF23 quartile: 90.3 RU/mL, interquartile range: 78.3–120.0) were more likely to be women with higher waist circumference, to have higher BMI and to be smokers. Increasing quartiles of FGF23 were associated with higher creatinine, PTH and CRP levels, but not 25(OH) D3 (Table 1). Regarding diet, participants in the upper FGF23 quartile had higher intakes of phosphorus, calcium, phosphorus to protein ratio and total energy intake (Table 2).

**Table 1:** Demographic, lifestyle, and clinical characteristics (potential predictors) of EPIC-Germany participants, overall and according to sex specific quartiles (Q) of FGF23 levels.

Characteristics	All	Q1	Q2	Q3	Q4
n	2134	533	534	533	534
Age, yrs ^a^	51.1 (50.7–51.5)	50.6 (49.9–51.3)	51.1 (50.3–51.8)	51.1 (50.3–51.8)	51.6 (50.9–52.4)
Body mass index, kg/m^2^ ^a^	26.1 (25.9–26.3)	25.7 (25.3–26.0)	26.3 (26.0–26.7)	26.0 (25.7–26.4)	26.6 (26.1–26.9)
*Waist circumference*, *cm* ^a^					
Men	95.6 (95.0–96.3)	94.3 (93.0–95.6)	96.2 (94.9–97.5)	95.7 (94.4–97.0)	96.3 (95.0–97.6)
Women	80.7 (80.0–81.4)	79.4 (78.1–80.7)	80.3 (79.0–81.7)	80.8 (79.5–82.1)	82.2 (80.9–83.4)
*Prevalent Comorbidities*, *%* ^b^					
Cardiovascular disease	3.3	3.0	3.4	3.2	3.7
Hypertension	33.8	33.0	32.8	32.3	37.1
Hyperlipidemia	31.6	29.3	32.2	33.4	31.4
Diabetes	3.7	3.6	4.1	3.0	4.3
High educational level, % ^b^	34.4	37.7	35.2	33.0	31.8
High physical activity, % ^b^	19.9	16.1	20.4	22.7	20.2
Smokers, % ^b^	21.0	18.8	17.0	21.2	27.0
*Markers* ^a^					
PTH, pg/mL	27.0 (18.1–39.0)	24.8 (26.3–26.0)	26.1 (24.9–27.4)	26.5 (25.3–27.9)	29.6 (28.2–31.1)
25(OH)D3, ng/mL	17.6 (12.5–23.5)	16.6 (16.0–17.2)	16.8 (16.2–17.5)	17.0 (16.3–17.6)	17.0 (16.3–17.7)
Creatinine, mg/dL	0.82 (0.82–0.83)	0.80 (0.78–0.82)	0.82 (0.81–0.84)	0.83 (0.81–0.84)	0.85 (0.83–0.87)
Total cholesterol, mg/dL	220.4 (218.0–222.7)	213.4 (209.0–218.2)	223.7 (219.0–228.4)	221.3 (216.7–225.9)	222.8 (218.1–227.5)
HDL, mg/dL	56.6 (55.8–57.4)	57.2 (55.7–58.7)	57.2 (55.7–58.7)	56.2 (54.7–57.7)	55.8 (54.3–57.3)

Data are expressed as:

^a^ Mean and 95% Confidence Interval (CI)

^b^ Percentage.

**Table 2:** Dietary characteristics (potential predictors) of EPIC-Germany participants according to sex specific quartiles (Q) of FGF23 levels.

Dietary variables	Q1	Q2	Q3	Q4
n	533	534	533	534
Median (IQR)^a^	31.2 (25.3–35.9)	46.5 (43.3–50.3)	61.5 (57.5–66.1)	90.3 (78.3–120.0)
*Macronutrients*, *%EI* ^b^				
Carbohydrate	43.9	44.4	44.8	44.5
Total protein	15.3	15.1	15.1	14.9
Animal protein	8.1	7.8	7.9	7.7
Plant protein	4.4	4.5	4.5	4.4
Fat	35.1	35.0	35.1	35.4
Fibre	2.2	2.2	2.2	2.1
Alcohol	5.7	5.5	5.0	5.2
*Minerals*, *mg/day* ^c^				
Phosphorus	1340 (1304–1375)	1327 (1291–1362)	1354 (1318–1389)	1396 (1361–1432)
Calcium	884.6 (854.6–914.6)	871.6 (841.7–901.5)	879.9 (850.0–909.9)	940 (910–970)
Potassium	3085(3011–3158)	3114 (3041–3188)	3132 (3059–3206)	3170.5 (3097.2–3243.9)
Magnesium	375 (366–384)	379 (370–388)	382 (372–391)	386 (377–395)
Phosphorus to protein ratio, mg/g	17.7 (17.5–17.9)	17.8 (17.6–18.0)	17.9 (17.7–18.1)	18.2 (18.0–18.5)
Intake calcium preparation, %^b^	3.8	4.5	5.6	5.1
Total energy intake, kcal/day	2027 (1973–2081)	2029 (1975–2083)	2056 (2002–2111)	2104 (2050–2158)

Data are expressed as:

^a^ Median and interquartile range (IQR)

^b^ Percentage

^c^ Mean and 95% Confidence Interval (CI).

The best subset of FGF23correlates is shown in Table 3. In the multivariable linear regression model, based on the best subset in which each predictor was mutually adjusted for each other, significant associations were observed between FGF23 and sex, smoking, PTH, creatinine, total cholesterol, CRP, intakes of calcium, iron, alcohol and total energy (Table 3).

**Table 3:** Multivariable linear regression for the associations between FGF23 concentrations and the best subset of FGF23 correlates, mutually adjusted for each other.

	Whole study population		Exclusion eGFR < 60 mL/min/1.73m^2^	
Parameter	β coefficient (95% CI)	P value^a^	β coefficient (95% CI)	P value^a^
	n = 2134		n = 2061	
Men	-0.21 (-0.28;-0.14)	<0.001	-0.22 (-0.30;-0.15)	<0.001
Physical activity, Q2	-0.0004 (-0.07;0.07)	0.99	-0.003 (-0.07;0.07)	0.93
Physical activity, Q3	-0.01 (-0.08;0.07)	0.89	-0.01 (-0.08;0.07)	0.89
Physical activity, Q4	0.07 (-0.01;0.15)	0.08	0.08 (-0.01;0.16)	0.07
Smokers	0.12 (0.06;0.18)	<0.001	0.12 (0.06;0.19)	<0.001
PTH, pg/mL^b^	0.13 (0.08;0.17)	<0.001	0.13 (0.08;0.16)	<0.001
Creatinine, mg/dL^b^	0.28 (0.14;0.41)	<0.001	0.27 (0.11;0.43)	0.001
Total cholesterol, mg/dL	0.001 (0.00003;0.001)	0.04	0.001 (0.0001;0.001)	0.01
HDL-cholesterol, mg/dL	-0.002 (-0.003;-0.0001)	0.06	-0.002 (-0.003;-0.0001)	0.04
C-reactive protein, mg/dL^b^	0.03 (0.01;0.05)	0.01	0.02 (-0.001;0.04)	0.07
Total protein intake, Q2	0.04 (-0.04;0.11)	0.37	0.02 (-0.06;0.10)	0.63
Total protein intake, Q3	-0.03 (-0.11;0.06)	0.41	-0.05 (-0.13;0.03)	0.21
Total protein intake, Q4	-0.04 (-0.12;0.05)	0.42	-0.05 (-0.15;0.04)	0.23
Calcium intake, mg/dL^b^	0.10 (0.03;0.18)	0.01	0.04 (-0.07;0.15)	0.45
Iron intake, mg/d^b^	-0.25 (-0.42;-0.08)	0.005	-0.29 (-0.47;-0.12)	0.001
Alcohol intake, g/d^b^	-0.02 (-0.04;-0.002)	0.03	-0.02 (-0.04;-0.001)	0.07
Energy intake, kcal/d^b^	0.25 (0.08;0.42)	0.003	0.21 (0.01;0.40)	0.03

eGFR stands for estimated glomerular filtration rate; Q2, Q3, Q4 stand for quartile 2, 3 and 4.

^a^ Based on mutual adjustment.

^b^ Natural log transformed.

Results from a multivariable logistic regression analysis indicate that men had a 66% lower probability and smokers a 64% higher probability of having FGF23 levels higher than 90 RU/mL (Table 4). Furthermore, each doubling in PTH, creatinine, and CRP was related to a higher probability of having increased FGF23. Among the dietary factors, each doubling in dietary calcium and total energy intake was related, respectively, to a 1.75 and to a 4.41 fold increased probability of having higher FGF23; in contrast, each doubling in iron intake was related to an 82% lower probability.

**Table 4:** Odd ratios (OR) and 95% Confidence Intervals (CI) for the associations between high FGF23 (> = 90 RU/mL) and the best subset of its significant correlates, mutually adjusted for each other.

	Whole study population		Exclusion eGFR < 60 mL/min/1.73m^2^	
Predictors	OR (95%CI)	P value^a^	OR (95%CI)	P value^b^
FGF23≥90 RU/mL	n = 269		n = 256	
Men	0.34 (0.24–0.49)	<0.001	0.28 (0.18–0.42)	<0.001
Smoking	1.64 (1.21–2.25)	0.002	1.62 (1.18–2.22)	0.003
PTH, pg/mL^c^	1.29 (1.10–1.50)	0.001	1.27 (1.08–1.49)	0.004
Creatinine, mg/dL^c^	2.17 (1.34–3.51)	0.002	2.29 (1.21–4.33)	0.01
C-reactive protein, mg/L^c^	1.16 (1.03–1.20)	0.005	1.10 (1.02–1.19)	0.01
Total cholesterol, mg/dL	1.00 (1.00–1.00)	0.47	1.00 (1.00–1.00)	0.82
Calcium intake, mg/d^c^	1.48 (1.11–1.96)	0.007	—	—
Iron intake, mg/d^c^	0.30 (0.16–0.57)	<0.001	0.30 (0.16–0.57)	<0.001
Alcohol intake, g/d^c^	0.94 (0.88–1.01)	0.07	0.95 (0.89–1.02)	0.17
Energy intake, g/d^c^	2.80 (1.50–5.20)	0.001	3.71 (2.03–6.79)	<0.001

^a^ Based on mutual adjustment for the whole study population (n = 2134).

^b^ Based on mutual adjustment after exclusion of participants with estimated glomerular filtration rate (eGFR) < 60 mL/min/1.73m^2^ (n = 2061).

^c^ Log base 2 transformed.

The same subset of FGF23 predictors was identified after exclusion of participants with eGFR <60 mL/min/1.73 m^2^, and results did not substantially change after multivariable linear and logistic regression analyses (Tables 3 and 4).

When the same analyses were repeated including the ratio between phosphorus and total protein as potential FGF23 predictor results were highly comparable to those reported in Table 3, with the dietary phosphorus to protein ratio emerging as a new predictor of FGF23 instead of calcium intake (S2 and S3 Tables).

## Discussion

In this cross-sectional analysis of middle-aged participants, sex, physical activity, smoking, PTH, creatinine, total- and HDL-cholesterol, CRP, total protein, calcium, iron, alcohol and total energy intake were identified as main potential predictors of FGF23. In particular, when these correlates were then studied in relation to FGF23 concentrations, mutually adjusted for each other, female sex, smokers, PTH, creatinine, and CRP, were all independently and positively associated with higher plasma FGF23 concentrations. In addition, intakes of calcium and total energy were positively related to higher FGF23 concentrations. Interestingly, iron intake was inversely related to higher FGF23.

Overall, our findings regarding positive associations between smoking, PTH, creatinine, CRP and higher FGF23 concentrations are in line with other epidemiologic studies conducted on the topic. However, these previous studies were mainly conducted in men [21, 36], elderly [8, 19, 36], or patients with coronary artery disease [8]. As such, the current study supports and generalizes the previous findings to a population of Caucasian middle aged men and women. In addition, the present findings expand our knowledge of FGF23 correlates by showing male sex and iron intake to be negatively related to higher FGF23 concentrations, and calcium and total energy to be positively related to higher FGF23 concentrations.

In line with another study, women had higher FGF23 levels than men [37]. Since increased FGF23 has been related to increased risk of major CVD, as also shown in our previous studies [12] [17], interestingly this finding may contribute to increase our understanding on why the risk of CVD in middle-aged women begins to approach the male risk [37]. Also smokers had higher FGF23 concentrations in our study population. There is no clear explanation on how smoking might influence FGF23 levels. However, a recent experimental study observed increased plasma FGF23 levels in mice after injection of cadmium [38]. Since smokers have markedly increased cadmium levels in their blood [39] this might explain the observed positive association with higher FGF23.

To the best of our knowledge, this is the first observational study reporting relationships between higher FGF23, and dietary intakes of calcium, iron, and total energy.

Calcium has been suggested to modulate FGF23 production [40]. The hypocalcemia observed in Wistar rats with normal kidney function, fed with a low-calcium/low–vitamin D diet, was associated with lower FGF23 levels. Interestingly, the same authors observed that an increase in dietary calcium intake in parathyroidectomized rats increased serum calcium and FGF23, decreased calcitriol levels and did not change the phosphorus levels [40]. Furthermore, in an open-label crossover study, subjects adhering to the high phosphate/calcium diet had increased serum FGF23 levels [41]. Overall, these findings suggest that calcium deficiency might reduce circulating FGF23 levels.

Despite high dietary phosphorus intake has been suggested to increase FGF23 levels [42, 43], in the present study it was not identified as a significant positive predictor. Though our findings are in line with those of the Chronic Renal Insufficiency Study [44], in which in stage 2–4 CKD patients no significant association was observed between FGF23 and dietary phosphorus intake, in the Health Professionals Follow-up Study each 500 mg increase in dietary phosphorus intake was associated with 3.4 RU/mL higher serum FGF23 levels[45]. Yet, whether a low dietary phosphorus diet can prevent FGF23 elevation is unknown since available evidence are inconsistent and unlikely conclusive. Also, no human clinical trials confirming or refuting this hypothesis exist [23]. Even so, whether FGF23 levels change after a dietary phosphate load, how this might occur is still not entirely understood [42, 46].

Besides calcium and phosphorus, an emerging area of investigation is represented by the potential regulatory role of iron on FGF23 levels [47, 48]. In the present study, each doubling in iron intake was related to an 82% lower probability of having higher FGF23 levels. The present finding supports the results of a recent study showing increased FGF23 levels in elderly subjects with low iron levels [49]. As reviewed by Bhattacharyya and colleagues [47], and later also by Gutierrez [50], there could be a clear involvement of iron in the FGF23 expression and/or secretion [51, 52].

To our knowledge, no studies so far have identified energy intake as a significant predictor of FGF23 concentrations. Although the role of energy intake in modulating FGF23 concentrations cannot be ruled out in this cross-sectional study, we could speculate that this finding might involve a dysregulation of α-klotho expression, the FGF23 regulating hormone and co-receptor [53], with a mechanism similar to that inducing klotho expression by calorie restriction [54].

Overall, these findings make the link between FGF23 and diet even more biologically plausible. Yet, further studies are warranted to determine the underlying mechanism of these relationships.

Limitations of our study include the cross-sectional design which does not enable determination of causality. FFQ may have underestimated the intake of phosphorus and protein. Hidden sources of phosphate added to foods in form of additives are usually not included in the food composition tables. However, considering that phosphorus is naturally found in protein-rich foods the ratio between phosphorus and protein has been suggested as a more appropriate method for estimating phosphorus intake from foods [35]. Besides, this method would ensure the computation of the protein content of a given food with the lowest possible phosphorus amount. Furthermore, this ratio has been found to be independent of the portion consumed and less influenced by systematic underestimation of total food consumption [55]. Interestingly, in a parallel analysis using the dietary phosphorus to protein ratio as FGF23 correlates we observed a 5.4 fold increased probability of having FGF23 levels higher than 90 RU/mL. Yet, this observation might add further strength to the hypothesized role played by dietary phosphorus intake on FGF23. Nevertheless, since large quantities of phosphate can originate from membrane phospholipids and in order to better estimate dietary phosphorus intake it would have been useful to analyse the amount of phosphate in the 24-h-urine. However, urine phosphate, as well as blood calcium and phosphorus levels, was not available in the present study. Another limitation is that data used for this investigation were collected about two decades ago; therefore nutritional intakes in particular may have changed since then. However, although absolute food intake may be different in the long run, the relation between dietary data and FGF23 concentrations is assumed to be similar over time. Finally, because this study was not designed to investigate biological mechanisms, further studies are necessary to understand at which level the identified correlates influence FGF23 concentrations.

## Conclusions

The observed associations between higher FGF23 and other traditional and non CVD risk factors may help to shed light on the possible pathophysiological mechanisms linking increased FGF23 to increased cardiovascular risk in the general population.

## Supporting Information

S1 Table Percentage and number of missing data in 27 variables used in imputation. (DOCX)

S2 Table Multivariable linear regression for the associations between FGF23 concentrations and the best subset of FGF23 correlates, including phosphorus to protein ratio, mutually adjusted for each other. (DOCX)

S3 Table Odd ratios (OR) and 95% Confidence Intervals (CI) for the associations between high FGF23 (> = 90 RU/mL) and the best subset of its significant correlates, including phosphorus to protein ratio, mutually adjusted for each other. (DOCX)
