# Supplementary material for: Potential Predictors of Plasma Fibroblast Growth Factor 23 Concentrations: Cross-Sectional Analysis in the EPIC-Germany Study
Source: PLoS One. 2015 Jul 20;10(7):e0133580. doi: 10.1371/journal.pone.0133580 (PMC4508099; doi:10.1371/journal.pone.0133580)
Supplement: S1 Table — (DOCX) [file pone.0133580.s001.docx]

**S1 Table.** Percentage and number of missing data in 27 variables used in imputation.

| **Variable** | **Degree of missingness** |  |
| --- | --- | --- |
|  | No. | % |
| Sex | 0 | — |
| Age | 0 | — |
| BMI | 13 | 0.60 |
| Smoking | 3 | 0.14 |
| Education | 0 | — |
| Physical activity | 0 | — |
| Hypertension | 0 | — |
| Hyperlipidemia | 1 | 0.05 |
| Diabetes | 0 | — |
| Prevalent CVD | 0 | — |
| Total cholesterol | 117 | 5.39 |
| HDL-cholesterol | 117 | 5.39 |
| C-reactive protein | 121 | 5.57 |
| FGF23 | 24 | 1.10 |
| PTH | 269 | 12.34 |
| 25(OH)D3 | 18 | 0.83 |
| Creatinine | 189 | 8.70 |
| Carbohydrate intake | 0 | — |
| Protein intake | 0 | — |
| Fat intake | 0 | — |
| Fibre intake | 0 | — |
| Alcohol intake | 0 | — |
| Calcium intake | 0 | — |
| Phosphorus intake | 0 | — |
| Potassium intake | 0 | — |
| Iron intake | 0 | — |
| Total energy intake | 0 | — |
