# Supplementary material for: Potential Predictors of Plasma Fibroblast Growth Factor 23 Concentrations: Cross-Sectional Analysis in the EPIC-Germany Study
Source: PLoS One. 2015 Jul 20;10(7):e0133580. doi: 10.1371/journal.pone.0133580 (PMC4508099; doi:10.1371/journal.pone.0133580)
Supplement: S2 Table — (DOCX) [file pone.0133580.s002.docx]

**S2 Table**. Multivariable linear regression for the associations between FGF23 concentrations and the best subset of FGF23 correlates, including phosphorus to protein ratio, mutually adjusted for each other.

| **Parameter** | **β coefficient (95% CI)** | **P value^a^** |
| --- | --- | --- |
| n=2134 |  |  |
| Men | -0.20 (-0.27;-0.13) | <0.001 |
| Physical activity, Q2 | -0.008 (-0.08;0.06) | 0.82 |
| Physical activity, Q3 | -0.02 (-0.09;0.06) | 0.69 |
| Physical activity, Q4 | 0.06 (-0.03;0.14) | 0.19 |
| Smokers | 0.12 (0.06;0.18) | <0.001 |
| PTH, pg/mL^b^ | 0.13 (0.08;0.17) | <0.001 |
| Creatinine, mg/dL^b^ | 0.26 (0.12;0.39) | <0.001 |
| Total cholesterol, mg/dL | 0.001 (0.00002;0.001) | 0.04 |
| HDL-cholesterol, mg/dL | -0.002 (-0.003;-0.0001) | 0.03 |
| C-reactive protein, mg/dL^b^ | 0.03 (0.01;0.05) | 0.01 |
| Phosphorus to protein ratio, mg/g | 0.02 (0.01;0.03) | <0.001 |
| Calcium supplement use | 0.11 (-0.01;0.23) | 0.08 |
| Iron intake, mg/d^b^ | -0.21 (-0.37;-0.04) | 0.01 |
| Alcohol intake, g/d^b^ | -0.03 (-0.04;-0.008) | 0.01 |
| Energy intake, kcal/d^b^ | 0.28 (0.12;0.44) | <0.001 |

Q2, Q3, Q4 stand for quartile 2, 3 and 4

^a^ Based on mutual adjustment.

^b^ Natural log transformed.
