# Supplementary material for: Potential Predictors of Plasma Fibroblast Growth Factor 23 Concentrations: Cross-Sectional Analysis in the EPIC-Germany Study
Source: PLoS One. 2015 Jul 20;10(7):e0133580. doi: 10.1371/journal.pone.0133580 (PMC4508099; doi:10.1371/journal.pone.0133580)
Supplement: S3 Table — (DOCX) [file pone.0133580.s003.docx]

**S3 Table**. Odd ratios (OR) and 95% Confidence Intervals (CI) for the associations between high FGF23 (>=90 RU/mL) and the best subset of its significant correlates, including phosphorus to protein ratio, mutually adjusted for each other.

| **Predictors** | **OR (95%CI)** | **P value^a^** |
| --- | --- | --- |
| FGF23≥90 RU/mL (n=269) |  |  |
| Men | 0.31 (0.21-0.46) | <0.001 |
| Smoking | 1.66 (1.21-2.27) | 0.002 |
| PTH, pg/mL ^b^ | 1.26 (1.08-1.47) | 0.003 |
| Creatinine, mg/dL ^b^ | 2.06 (1.27-3.36) | 0.004 |
| C-reactive protein, mg/L ^b^ | 1.12 (1.04-1.20) | 0.004 |
| Total cholesterol, mg/dL | 1.00 (1.00-1.00) | 0.59 |
| HDL-cholesterol, mg/dL | 0.99 (0.98-1.00) | 0.15 |
| Phosphorus to protein ratio, mg/g ^b^ | 5.35 (2.69-10.6) | <0.001 |
| Iron intake, mg/d ^b^ | 0.37 (0.20-0.69) | 0.002 |
| Alcohol intake, g/d ^b^ | 0.93 (0.87-1.00) | 0.04 |
| Energy intake, g/d ^b^ | 3.25 (1.80-5.87) | <0.001 |

Q2, Q3, Q4 stand for quartile 2, 3 and 4

^a^Based on mutual adjustment.

^b^ Log base 2 transformed.
